# Facile synthesis of palladium nanoparticles on hierarchical hollow silica spheres and its catalytic properties in Suzuki-reaction

**DOI:** 10.1098/rsos.180545

**Published:** 2018-09-12

**Authors:** Mao Liu, Liuli Wu, Jianjie Han, Xiuhong Xu, Chengyuan He, Peng Wang, Qian Wei, Wanliang Yang

**Affiliations:** Department of Chemistry, College of Chemistry and Chemical Engineering, Guizhou University, Guiyang, Guizhou 550025, People's Republic of China

**Keywords:** heterogeneous catalysis, hierarchical hollow silica spheres, palladium nanoparticles

## Abstract

A novel well-dispersed Pd@HHSS catalyst was synthesized by reduction of Pd(OAc)_2_ immobilized on HHSS we reported. When the ratios of Pd/SiO_2_ were 5 : 100 and 10 : 100, the Pd nanoparticles size was about 5–10 nm. The Pd@HHSS catalyst (Pd/SiO_2_ = 10 : 100) showed high catalytic activity in Suzuki-reaction with yields of 91–99% and the catalyst is reusable after four successive cycles without obvious loss of catalytic activity.

## 1. Introduction

The transition metal catalytic formation of C–C bonds plays an unparalleled role in organic synthesis, and the catalysts as the most important centre of the reaction have been wildly studied. Though homogeneous transition metal catalysts, especially palladium [1–4], have exhibited great catalytic activity in catalytic reactions [5–7], they suffered from separation, reusability and toxicity problems. Therefore, homogeneous catalysts are limitedly used in industry [8–10]. Though the catalytic activity of heterogeneous catalysts is generally inferior to that of their homogeneous counterparts, heterogeneous catalysts often benefit from the reusability of the catalysts.

Over the last decades, transition metal nanoparticles with large surface area and heterogeneous catalytic properties attract a lot of chemists [10–17]. Up to now, a vast number of the transition metal nanoparticles have been obtained by reduction of corresponding metal ions in the presence of stabilizers [18–22], and their catalytic performance mostly depend on their active species and stabilizers [23,24]. Palladium, as one of the most studied transition metals in homogeneous catalysis, undoubtedly has been used for synthesis of nanoparticles with some stabilizers, which exhibit high activity in some reactions [25–31]. Various stabilizers such as metal oxides [32,33], zeolites [34], carbon structures [35], polymers [36], mesoporous silicas [37,38] etc. were used to immobilize the Pd-NPs. Owing to their high specific surface area, strong adsorption capacity, good hydrothermal stability and low cost, ordered mesoporous silica materials (e.g. SBA-15, KCC-1, HHSS) were used as novel potential supports [39–40].

Recently, we have developed new high surface area silica nanospheres (HHSS) with excellent textural properties (high surface area, pore volume and pore size). HHSS displays high surface area and accessibility owing to its unprecedented small-hollow-sphere@large-hollow-sphere morphology, which affords high performance as a catalyst support [41], and we want to examine the performance of HHSS as a stabilizer to immobilize palladium in preparation of nanoparticles. In this paper, we wish to report our recent studies on synthesis of well-dispersed novel palladium nanoparticles supported on HHSS even at high noble-metal loading (Pd@HHSS, the molar ratios of Pd/SiO_2_ = 5 : 100, 10 : 100 and 20 : 100) and its catalytic properties in Suzuki-reaction.

## 2. Material and methods

### 2.1. Materials and preparation

Tetraethyl orthosilicate (TEOS, A.R), aqueous ammonia solution (28 wt%) and ethanol (A.R) were purchased from Xilong Chemical Co., Ltd (Guangdong, China). *n*-octane (A.R), cetyltrimethylammoniumbromide (CTAB, A.R) and reactive brilliant red (RBR, A.R) were obtained from Tianjin Fuchen Chemical Reagents Factory (Tianjin, China). The aryl bromides (A.R), arylboronic acids (A.R), NaBH_4_ (A.R) and Pd(AcO)_2_ (A.R) were purchased from J&K Scientific Ltd (Beijing, China). All the reagents were used without further purification.

#### 2.1.1. Synthesis of hierarchical hollow silica spheres

Hierarchical hollow silica spheres (HHSS) were prepared in an alkaline solution according to our previous research [41]. In a typical synthesis, 1.38 g CTAB was dissolved in a mixture of 66 ml H_2_O and 14 ml ammonia (1 mol l^−1^) to make an emulsion, and then 20 ml *n*-octane was added. After stirring for 0.5 h, 7.2 ml TEOS was added dropwise into the mixture, followed by stirring for another 0.5 h. Then, the mixture was transferred into a Teflon-lined autoclave and heated at 373 K for 24 h under autogenous pressure. The sample was separated by filtration, washed with deionized water and dried at 333 K for 6 h. The as-synthesized solid sample was collected and calcined at 823 K for 6 h in air to remove CTAB and other organic components.

#### 2.1.2. Synthesis of Pd@HHSS

Pd@HHSS composites with the different molar ratios of Pd/SiO_2_ = 5 : 100, 10 : 100 and 20 : 100 were synthesized. For example of Pd/SiO_2_ (10 : 100): to a stirred solution of Pb(OAc)_2_ (89.8 mg, 0.40 mmol) in water (20 ml) was added HHSS (240.3 mg, 4 mmol based on SiO_2_). The mixture was stirred for 0.5 h to form a brown suspension, and then a solution of NaBH_4_ (75.7 mg, 2.00 mmol) in EtOH (20 ml) was added. Immediately the colour of the suspension turned to black. After stirring for 3 h, the product was obtained as a black powder by centrifugation, washed with EtOH twice and dried at 70°C overnight.

### 2.2. Characterizations

X-ray diffraction (XRD) patterns were recorded on a Rigaku D/Max 2500 VBZ+/PC diffractometer using Cu-Kα radiation (*λ* = 0.1541 nm). N_2_ adsorption–desorption isotherms were obtained using a Micromeritics ASAP2020M instrument. The materials were pretreated in vacuum at 573 K for 6 h. The specific surface area (*S*_BET_) was estimated using Brunauer–Emmett–Teller (BET) equation. The pore size distribution was calculated from the desorption branch of the isotherm using Barrett–Joyner–Halenda (BJH) method. The morphology of the samples was examined by high-resolution transmission electron microscope (HRTEM) on a JEM-3010 with an accelerating voltage of 200 kV. The scanning electron microscope (SEM) photographs of the samples were obtained using a Hitachi S-4700 electron microscope.

### 2.3. Suzuki-reaction

To a tube charged with aryl bromides (1.5 mmol), aryl boronic acids (1.8 mmol), Na_2_CO_3_ (477.0 mg, 4.5 mmol) and Pd@HHSS (0.0075 mmol Pd) was added solvent (3.0 ml EtOH and 3.0 ml water). The mixture was stirred at 75°C till the reaction was completed, and then extracted with CH_2_Cl_2_ (3 × 30 ml) and dried with Na_2_SO_­4_. After filtration, the mixture was concentrated and purified by flash chromatography to give the corresponding coupling product.

## 3. Results and discussion

**Scheme 1.**
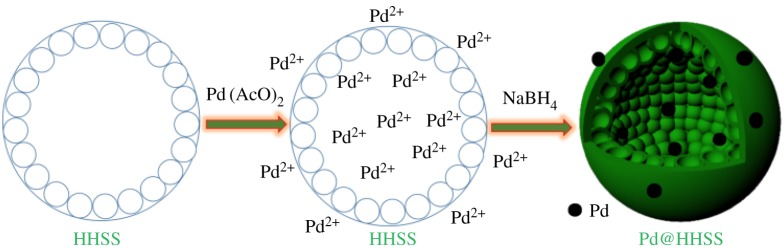
Synthesis of palladium nanoparticles immobilized on HHSS.

The HHSS with high BET surface area (726 m^2^ g^−1^), good stability and large diameter (90–150 nm) can be attractive for immobilization of Pd nanoparticles by exploiting both their exterior and hollow interior. It is to be believed that Pd(OAc)_2_ is ionized to Pd(II) oxide species (Pd-O) at the surface of the HHSS during the impregnation step, and could be further reduced to Pd nanoparticles and immobilized on HHSS during the reduction step. Based on these considerations, nanoparticles were synthesized by reduction of Pd(AcO)_2_ in suspension of HHSS with NaBH_4_ as reductant (scheme 1).

**Figure 1.**
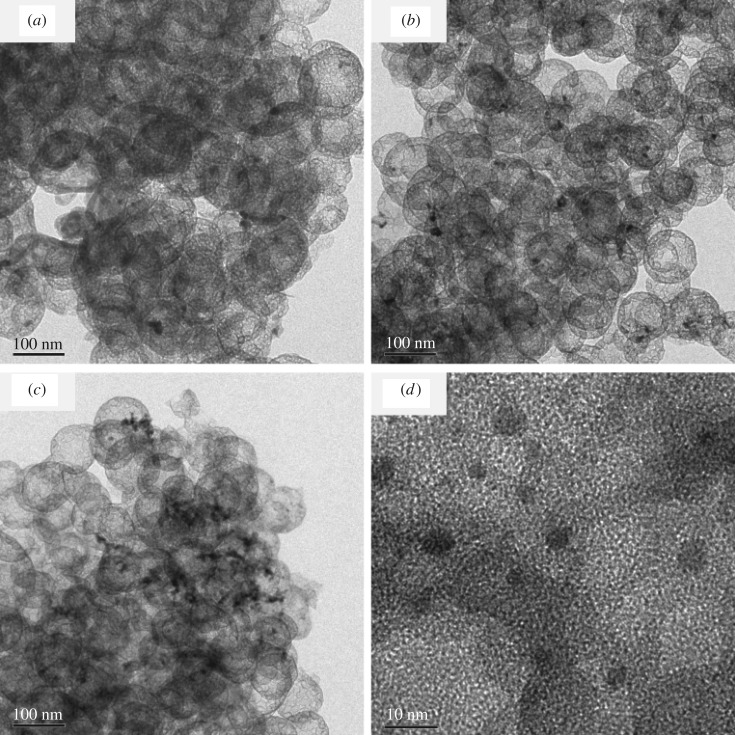
Transmission electron microscopic images of Pd@HHSS, the molar ratio of 5 : 100 (*a*), 10 : 100 (*b*,*d*) and 20 : 100 (*c*).

The morphology of Pd@HHSS with different molar ratios of Pd/SiO_2_ (5 : 100, 10 : 100 and 20 : 100) was studied by HRTEM (as shown in figure 1). The HHSS is constructed of spherical particles with a diameter between 90 and 150 nm having a small-ball@big-ball structure. The shell of the big hollow sphere is composed of small hollow spheres (20 nm), and the small hollow spheres are covered with an external wall. As shown in figure 1, most of the hollow spheres remained spherical and intact with smooth outer surface. When the ratios of Pd/SiO_2_ were 5 : 100 and 10 : 100, the Pd nanoparticles were well dispersed without obvious aggregation (figure 1*a,b*). The size of Pd nanoparticles was about 5–10 nm (figure 1*d*). When Pd/SiO_2_ = 20 : 100, the Pd nanoparticles tended to form aggregated metal clusters rather than well-dispersed nanoparticles (figure 1*c*). The well-dispersed nanoparticles were of great significance in adsorbing the reactants during the Suzuki-reaction process, so the ratio of Pd/SiO_2_ should not be up to 20 : 100.

**Figure 2.**
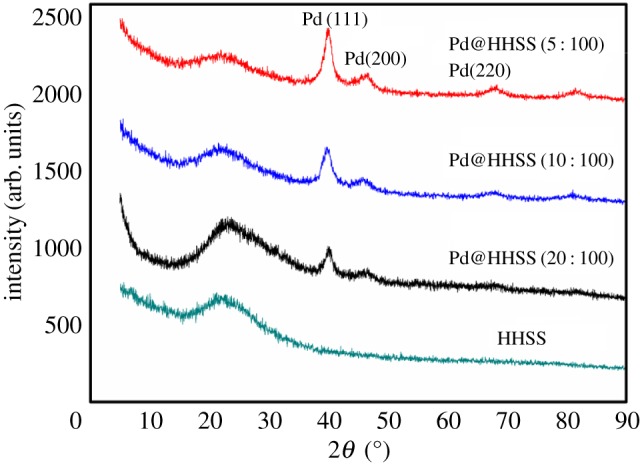
XRD patterns of Pd@HHSS, the molar ratio of Pd/SiO_2_ = 5 : 100, 10 : 100 and 20 : 100.

The phase purity and crystallinity of the HHSS and Pd@HHSS were checked by XRD (as shown in figure 2). The broad peak centred at 2*θ* = 23° was due to amorphous silica of HHSS. The peaks at 2*θ* = 40.1°, 46.7° and 68.1° all agree with the standard Pd structure (JCPDS no. 046–1043). The particle size (8.4 nm) of Pd nanoparticles on Pd@HHSS (Pd/SiO_2_ = 10 : 100) was measured by Scherrer equation according to the XRD pattern, showing good correlation with the HRTEM images (5–10 nm).

**Figure 3.**
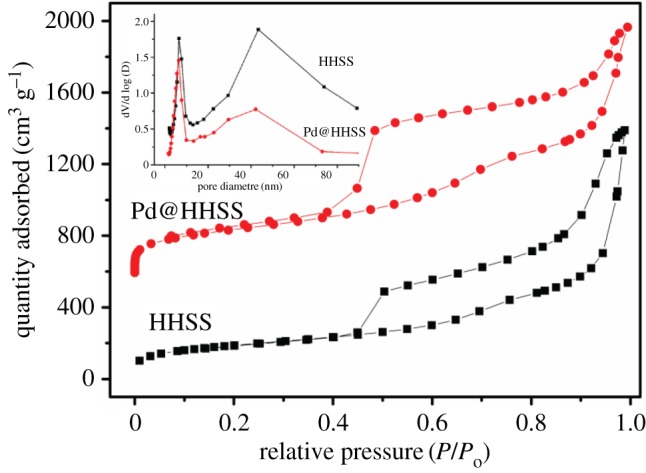
Nitrogen absorption–desorption isotherms for HHSS and Pd@HHSS (Pd/SiO_2_ = 10 : 100) with pore size distributions in the inset.

N_2_ adsorpion–desorption isotherms of the HHSS and Pd@HHSS (Pd/SiO_2_ = 10 : 100) are given in figure 3. Both isotherms exhibited a typically type IV pattern (IUPAC) with two capillary condensation steps occurring in the high relative pressure range of 0.7–0.8 and 0.9–0.95, indicative of bimodal large nanopores which may be attributed to the voids from the aggregation or random packing of hollow spheres [41]. The nitrogen uptake in the condensation step of Pd@HHSS at *P*/*P*_0_ = 0.9–1.0 increased slower than that of the HHSS, suggesting that the voids decreased because of Pd nanoparticles. Pd loading partially blocked the hollow silica spheres, leading to the surface area (533 m^2^ g^−1^) and pore volume (1.24 cm^3^ g^−1^) of Pd@HHSS both being lower than those of HHSS (726 m^2^ g^−1^ and 2.15 cm^3^ g^−1^). This could be verified by the pore size distribution as shown in the inset of figure 3.

**Figure 4.**
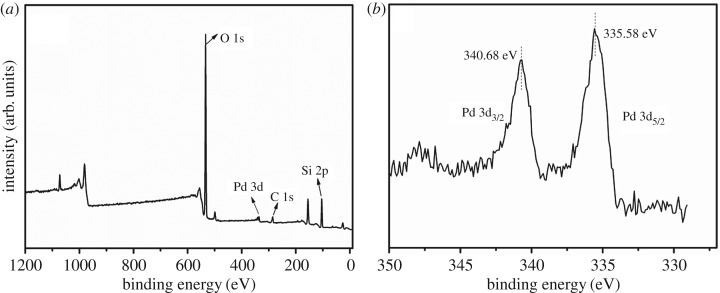
XPS spectra for the as-prepared Pd@HHSS (Pd/SiO_2_ = 10 : 100). (*a*) Full XPS spectra, (*b*) Pd3d states.

As shown in figure 4*a*, full XPS spectra consist of Pd, Si, O and C elements. Among them, C can be excluded from the product as base materials. The Pd 3d high-resolution XPS (figure 4*b*) displays two peaks at 340.68 and 335.58 eV, which are attributed to Pd^0^ nanoparticles with the peaks of Pd 3d in Pd@HHSS [42,43].

The Suzuki-reaction was first carried out with 4-bromoanisole (1.5 mmol) and phenylboronic acid (1.8 mmol) as the substrates and the Pd@HHSS (0.0075 mmol Pd) with different mole ratios of Pd/SiO_2_ = 5 : 100, 10 : 100 and 20 : 100 as catalysts. The conversions of 4-bromoanisole were raised from 73% to 91% as Pd/SiO_2_ increased from 5 : 100 to 10 : 100 at 75°C for 1 h determined by ^1^H NMR of the crude reaction mixture based on 4-bromoanisole. However, the conversion was only 5% when Pd/SiO_2_ = 20 : 100 under the same condition. This can be explained that Pd nanoparticles are well dispersed on the Pd@HHSS (Pd/SiO_2_ = 5 : 100 and 10 : 100), but Pd nanoparticles are aggregated to form bigger particles when Pd/SiO_2_ = 20 : 100. Consideration of the activity and the amount of HHSS used, the Pd@HHSS (Pd/SiO_2_ = 10 : 100) was chosen to be further studied.

The Suzuki-reaction was carried out with aryl bromides and aryl boronic acids as the substrates and the Pd@HHSS (Pd/SiO_2_ = 10 : 100) as the catalyst. As shown in table 1, the coupling reaction can be applied to a wide variety of aryl bromides and aryl boronic acids to give the corresponding coupling products with yields of 91–99%, and the amount of the catalyst can fall to 0.5% equivalent. The catalytic activity can be affected by the electronic effects of the substituents, but the yields were still above 91%.

**Table 1.** Suzuki-reaction on Pd@HHSS (Pd/SiO_2_ = 10 : 100).^a^

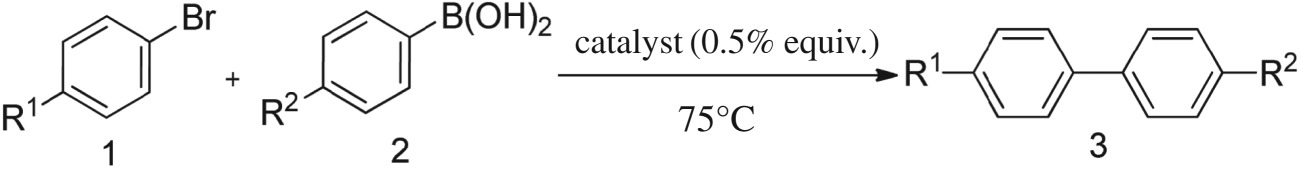				
entry	aryl bromide (**1**)	arylboronic acid (**2**)	time (h)	yield^b^ (%)
1	a, *R*^1^ = Me	a, *R*^2^ = H	6	98
2	b, *R*^1^ = MeO	b, *R*^2^ = H	15	91
3	c, *R*^1^ = Cl	c, *R*^2^ = H	18	99
4	d, *R*^1^ = CN	d, *R*^2^ = H	5	94
5	e, *R*^1^ = MeO	e, *R*^2^ = Me	22	91
6	f, *R*^1^ = Cl	f, *R*^2^ = Me	2	99
7	g, *R*^1^ = CN	g, *R*^2^ = Me	5	99
8	h, *R*^1^ = Me	h, *R*^2^ = MeO	20	91
9	i, *R*^1^ = MeO	i, *R*^2^ = MeO	15	95
10	j, *R*^1^ = Cl	j, *R*^2^ = MeO	5	97

^a^All reactions were carried out with aryl bromides (1.50 mmol), aryl boronic acids (1.50 mmol) and Pd@HHSS (Pd/SiO_2_ = 10 : 100) (5.3 mg, 0.0075 mmol Pd) in solvent (3 ml EtOH and 3 ml water).

^b^Isolated yields based on aryl bromides.

We have tested the reusability of the catalyst, and it was investigated with p-bromotoluene and phenylboronic acid as the substrates by carrying out four consecutive cycles using the same reaction conditions. After each run, the catalyst was recovered by filtration. The catalytic activity did not change considerably after four cycles. The conversion of the fourth catalytic reaction was still 95% (determined by ^1^H NMR).

## 4. Conclusion

HHSS are attractive for immobilization of Pd nanoparticles by exploiting both their exterior and hollow interior. Pd nanoparticles supported on HHSS (the mole ratios of Pd/SiO_2_ = 5 : 100, 10 : 100 and 20 : 100) were synthesized. When the mole ratios of Pd/SiO_2_ = 5 : 100 and 10 : 100, the Pd nanoparticles (5–10 nm) are well dispersed, while the Pd nanoparticles tend to form aggregated metal clusters when Pd/SiO_2_ = 20 : 100. Pd loading partially blocks the hollow silica spheres, leading to decreasing of the surface area and pore volume. The Pd@HHSS (Pd/SiO_2_ = 10 : 100) showed high catalytic activity in Suzuki-reaction with yields of 91–99%. It is easy to recover the catalysts after filtration and the catalysts are reusable after four successive cycles without obvious loss of catalytic activity.

## Supplementary Material

Pd@HHSS (SM)

## Data accessibility

The datasets supporting this article have been uploaded as part of the electronic supplementary material.
