# Supplementary material for: Facile synthesis of palladium nanoparticles on hierarchical hollow silica spheres and its catalytic properties in Suzuki-reaction
Source: R Soc Open Sci. 2018 Sep 12;5(9):180545. doi: 10.1098/rsos.180545 (PMC6170588; doi:10.1098/rsos.180545)
Supplement: Pd@HHSS (SM) [file rsos180545supp1.pdf]

Facile synthesis of palladium nanoparticles on hierarchical hollow silica spheres (HHSS) and its catalytic properties in Suzuki-reaction

Mao Liu, *[a] Liu-Li Wu,[a] Jian-Jie Han,[a] and Wanliang Yang*[a]

[a] *Department of Chemistry, College of Chemistry and Chemical Engineering, Guizhou University, Guiyang, Guizhou Province 550025, PR China*

Supplementary Information

Table of Contents

Experimental procedures and characterization data S-2

NMR spectra S-5

Table 1, entry 1

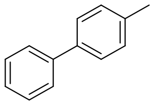

3a[1]

White solid ; IR (KBr) 2916, 1519 cm^{-1}; ^{1}H NMR (400 MHz, $CDCl_3$) δ 7.64-7.55 (m, 2H), 7.53-7.47 (m, 2H), 7.47-7.40 (m, 2H), 7.37-7.30 (m, 1H), 7.26 (d, J = 8.0 Hz, 2H), 2.41 (s, 3H); ^{13}C NMR (100 MHz, $CDCl_3$) δ 141.3, 138.5, 137.2, 129.7, 128.9, 127.2, 21.3; mp 48-49 ℃ .

Table 1, entry 2

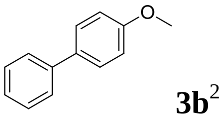

3b[2]

White solid ; IR (KBr) 3000, 1653, 1250 cm^{-1}; ^{1}H NMR (400 MHz, $CDCl_3$) δ 7.61-7.49 (m, 4H), 7.46-7.38 (m, 2H), 7.34-7.28 (m, 1H), 7.04-6.93 (m, 2H), 3.86 (s, 3H); ^{13}C NMR (100 MHz, $CDCl_3$) δ 159.3, 141.0, 134.0, 128.9, 128.4, 127.0, 126.9, 114.4, 55.6; mp 88-89 ℃ .

Table 1, entry 3

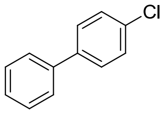

3c[2]

White solid ; IR (KBr) 3057, 1655 cm^{-1}; ^{1}H NMR (400 MHz, $CDCl_3$) δ 7.62-7.49 (m, 4H), 7.49-7.33 (m, 5H); ^{13}C NMR (100 MHz, $CDCl_3$) δ 140.2, 139.8, 133.5, 129.1, 128.6, 127.8, 127.2; mp 79-81 ℃ .

Table 1, entry 4

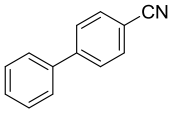

3d[2]

White solid ; IR (KBr) 2224, 1603 cm^{-1}; ^{1}H NMR (400 MHz, $CDCl_3$) δ 7.90-7.64 (m, 4H), 7.59 (d, J = 8.0 Hz, 2H), 7.53-7.46 (m, 2H), 7.46-7.39 (m, 1H); ^{13}C NMR (100 MHz, $CDCl_3$) δ 145.9, 139.4, 132.8, 129.3, 128.9, 127.9, 127.4, 119.2, 111.1; mp 83-84 ℃.

Table 1, entry 5 or **Table 1, entry 8**

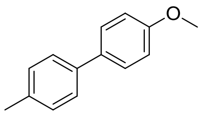

3e[3] or **3h**[3]

White solid; IR (KBr) 2967, 1639, 1251 cm^{-1}; ^1H NMR (400 MHz, $CDCl_3$) δ 7.56-7.48 (m, 2H), 7.48-7.40 (m, 2H), 7.23 (d, *J* = 8.0 Hz, 2H), 7.02-6.90 (m, 2H), 3.85 (s, 3H), 2.39 (s, 3H); ^{13}C NMR (100 MHz, $CDCl_3$) δ 159.1, 138.1, 136.6, 133.9, 129.6, 128.2, 126.8, 114.3, 55.5, 21.3; mp 110-111 ℃ .

Table 1, entry 6

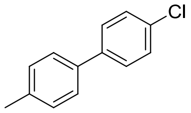

3f[3]

White solid ; IR (KBr) 2916, 1651 cm^{-1}; ^1H NMR (400 MHz, $CDCl_3$) δ 7.55-7.49 (m, 2H), 7.48-7.43 (m, 2H), 7.42-7.36 (m, 2H), 7.26 (d, *J* = 8.0 Hz, 2H), 2.40 (s, 3H); ^{13}C NMR (100 MHz, $CDCl_3$) δ 139.8, 137.7, 137.3, 133.2, 129.8, 129.0, 128.4, 127.0, 21.3; mp 121-122 ℃ .

Table 1, entry 7

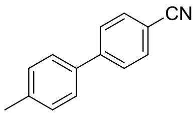

3g[4]

White solid ; IR (KBr) 2920, 2223, 1603 cm^{-1}; ^1H NMR (400 MHz, $CDCl_3$) δ 7.77-7.61 (m, 4H), 7.53-7.44 (m, 2H), 7.29 (d, *J* = 8.0 Hz, 2H), 2.42 (s, 3H); ^{13}C NMR (100 MHz, $CDCl_3$) δ 145.7, 138.9, 136.4, 132.7, 130.0, 127.6, 127.2, 119.2, 110.6, 21.4; mp 107-109 ℃.

Table 1, entry 9

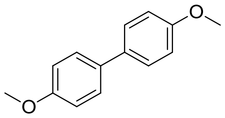

3i[5]

White solid ; IR (KBr) 2957, 1653, 1249 cm^{-1}; ^1H NMR (400 MHz, $CDCl_3$) δ 7.53-7.42 (m, 4H); 7.01-6.91 (m, 4H); 3.85 (s, 6H); ^{13}C NMR (100 MHz, $CDCl_3$) δ 158.8, 133.6, 127.9, 114.3, 55.5; mp 179-180 ℃.

Table 1, entry 10

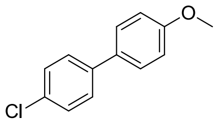

3j[5]

White solid ; IR (KBr) 2962, 1653, 1261 cm^{-1}; ^{1}H NMR (400 MHz, $CDCl_3$) δ 7.54-7.43 (m, 4H); 7.43-7.33 (m, 2H); 7.02-6.92 (m, 2H); 3.85 (s, 3H); ^{13}C NMR (100 MHz, $CDCl_3$) δ 159.5, 139.4, 132.8, 132.6, 129.0, 128.2, 128.1, 114.5, 55.6; mp 113-115 ℃.

The ^{1}H and ^{13}C spectra were in accordance with those described in literatures.

1. M. Bullpitt and W. Kitching, *J. Org. Chem.*, 1976, **41**, 5.
2. L. Bai and J.-X. Wang, *Adv. Synth. Catal.*, 2008, **350**, 315.
3. Z. Xiong, N. Wang, M. Dai, A. Li, J. Chen and Z. Yang, *Org. Lett.*, 2004, **6**, 19.
4. D.-G. Yu, M. Yu, B.-T. Guan, B.-J. Li, Y. Zheng, Z.-H. Wu, and Z.-J. Shi, *Org. Lett.*, 2009, **11**, 15,
5. I. Cepanec, M. Litvic, J. Udikovic, I. Pogorelic and M. Lovric, *Tetrahedron*, 2007, **63**, 5614.

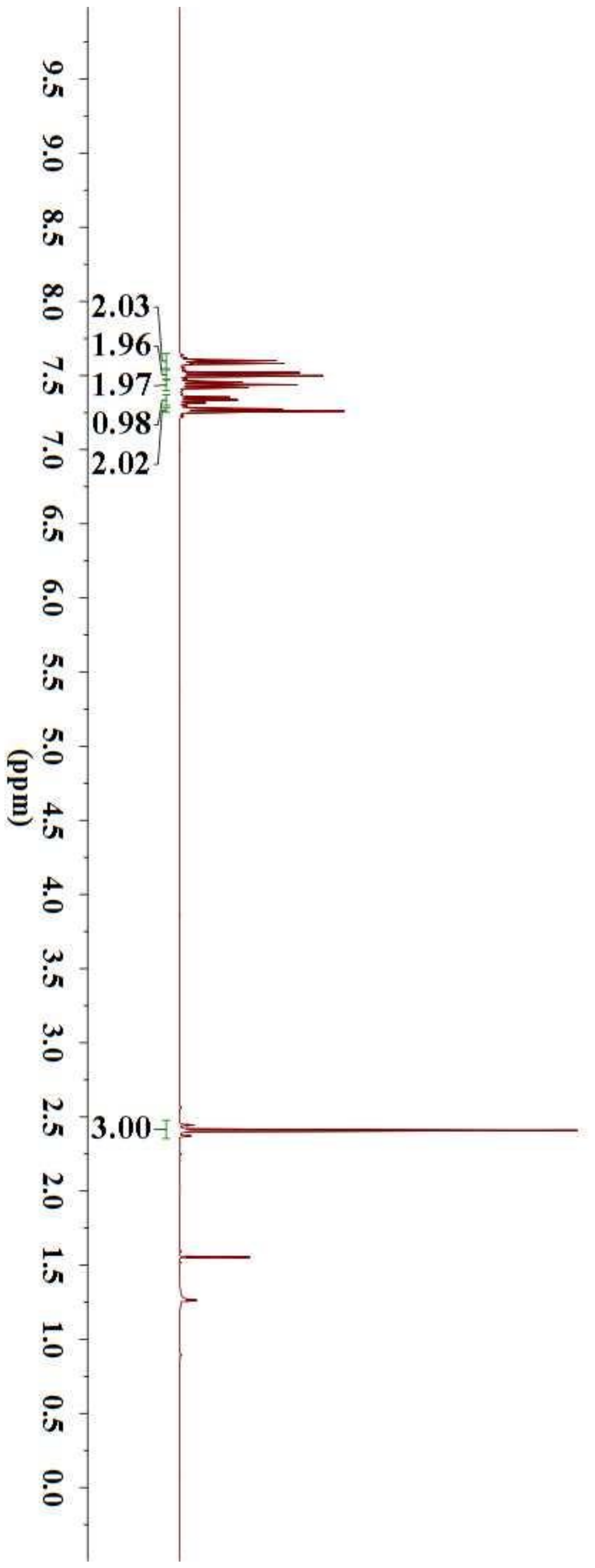
9.5 9.0 8.5 8.0 7.5 7.0 6.5 6.0 5.5 5.0 4.5 4.0 3.5 3.0 2.5 2.0 1.5 1.0 0.5 0.0
(ppm)
2.03
1.96
1.97
0.98
2.02
3.00

3a

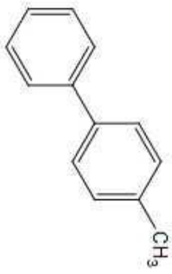
CH3

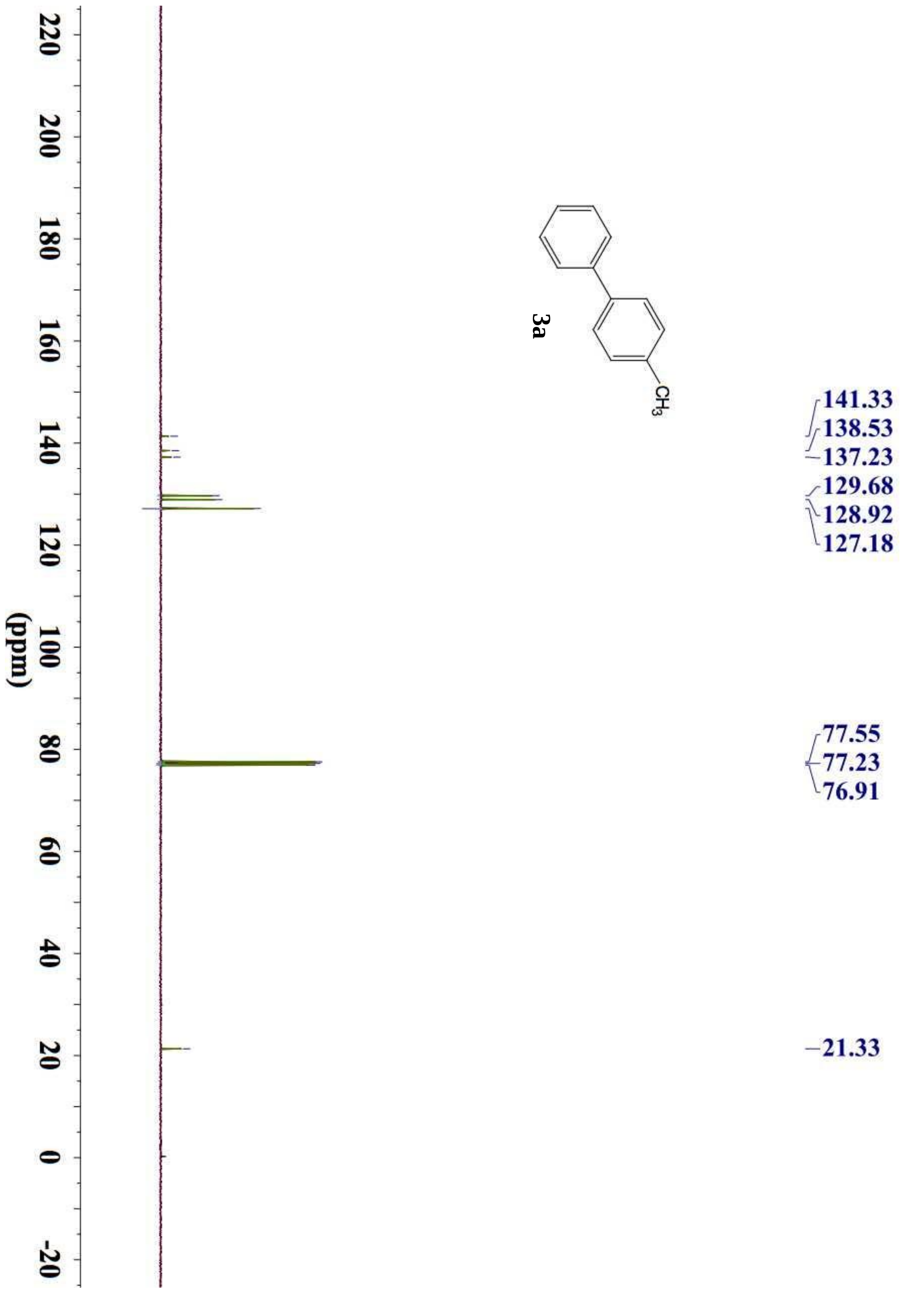
3a
CH3
141.33
138.53
137.23
129.68
128.92
127.18
77.55
77.23
76.91
21.33
220
200
180
160
140
120
100
80
60
40
20
0
-20
(ppm)

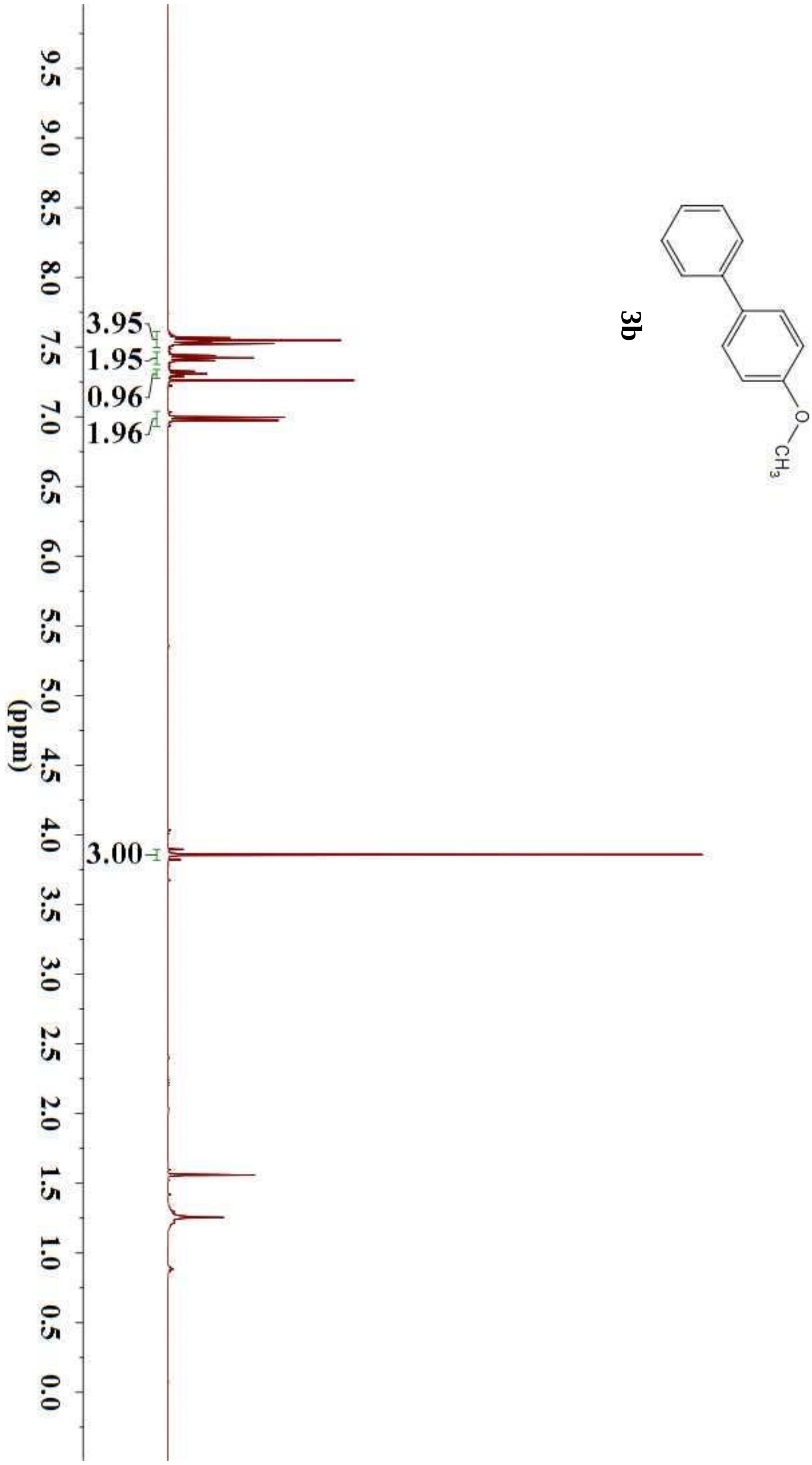
3b
O
CH3
3.95
1.95
0.96
1.96
3.00
9.5
9.0
8.5
8.0
7.5
7.0
6.5
6.0
5.5
5.0
4.5
4.0
3.5
3.0
2.5
2.0
1.5
1.0
0.5
0.0
(ppm)

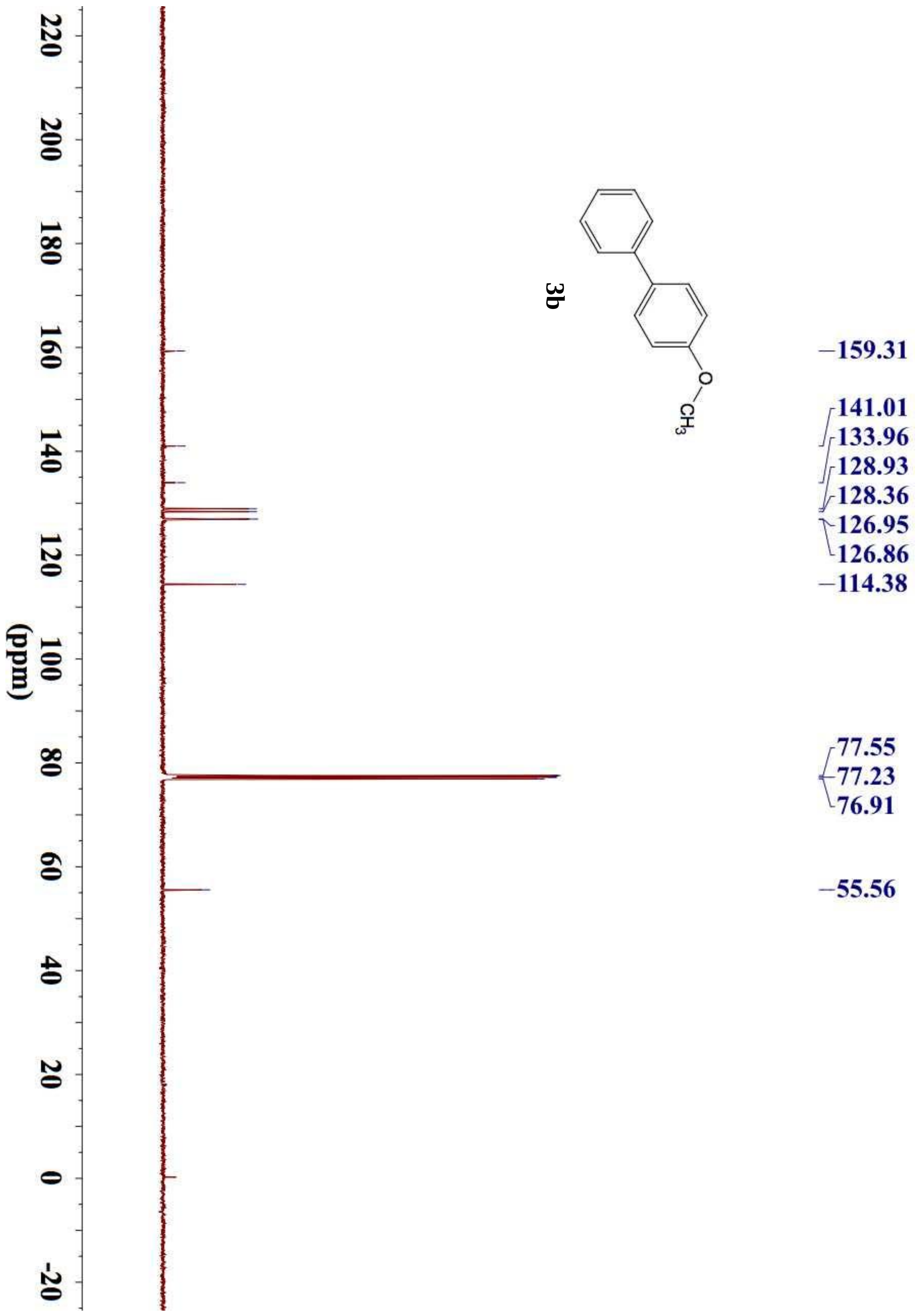
3b
O
CH3
159.31
141.01
133.96
128.93
128.36
126.95
126.86
114.38
77.55
77.23
76.91
55.56
220
200
180
160
140
120
100
80
60
40
20
0
-20
(ppm)

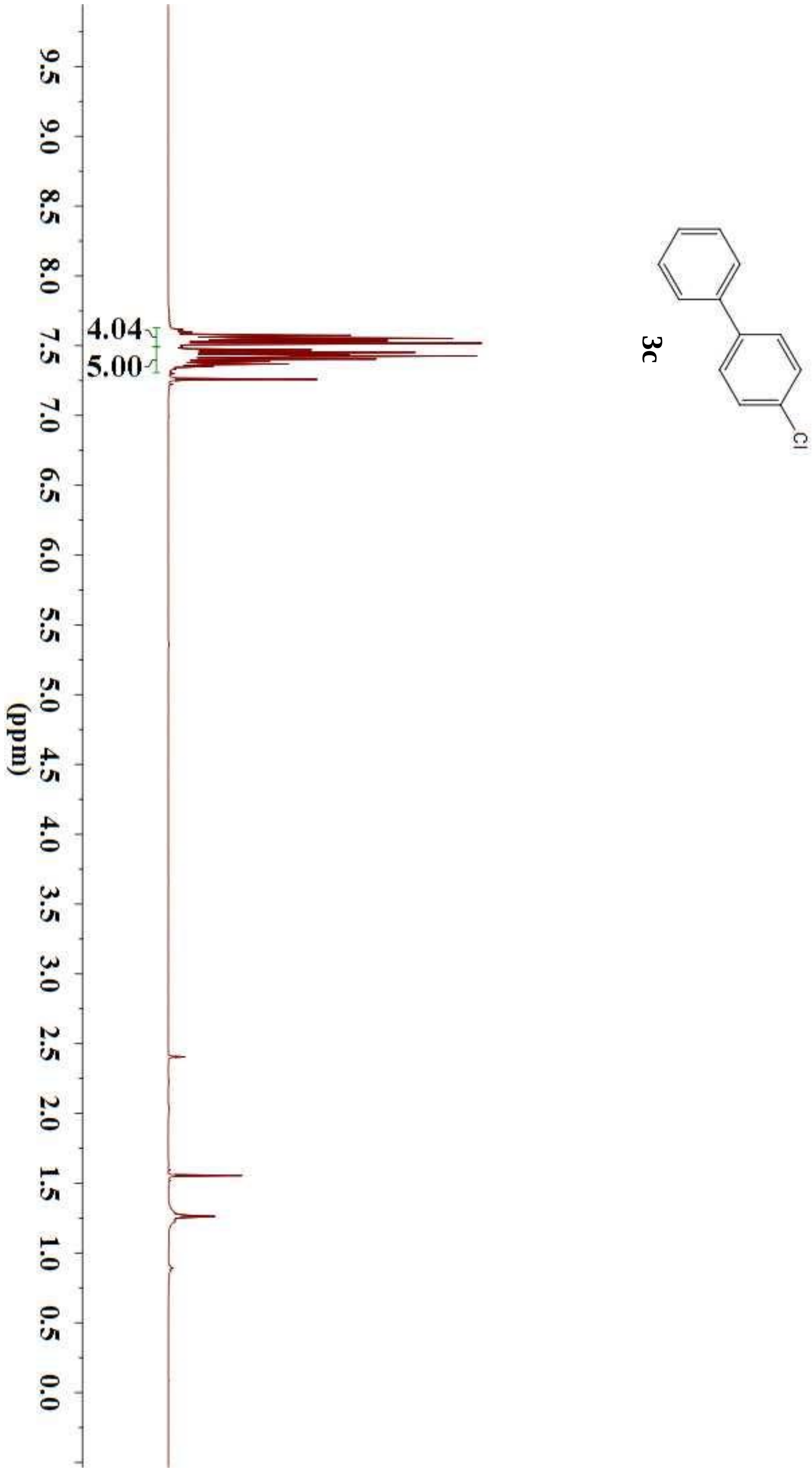

9.5
9.0
8.5
8.0
7.5
7.0
6.5
6.0
5.5
5.0
4.5
4.0
3.5
3.0
2.5
2.0
1.5
1.0
0.5
0.0
(ppm)
4.04
5.00
Cl
3c

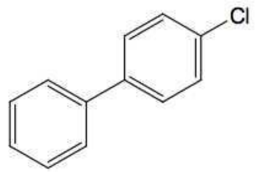

3c

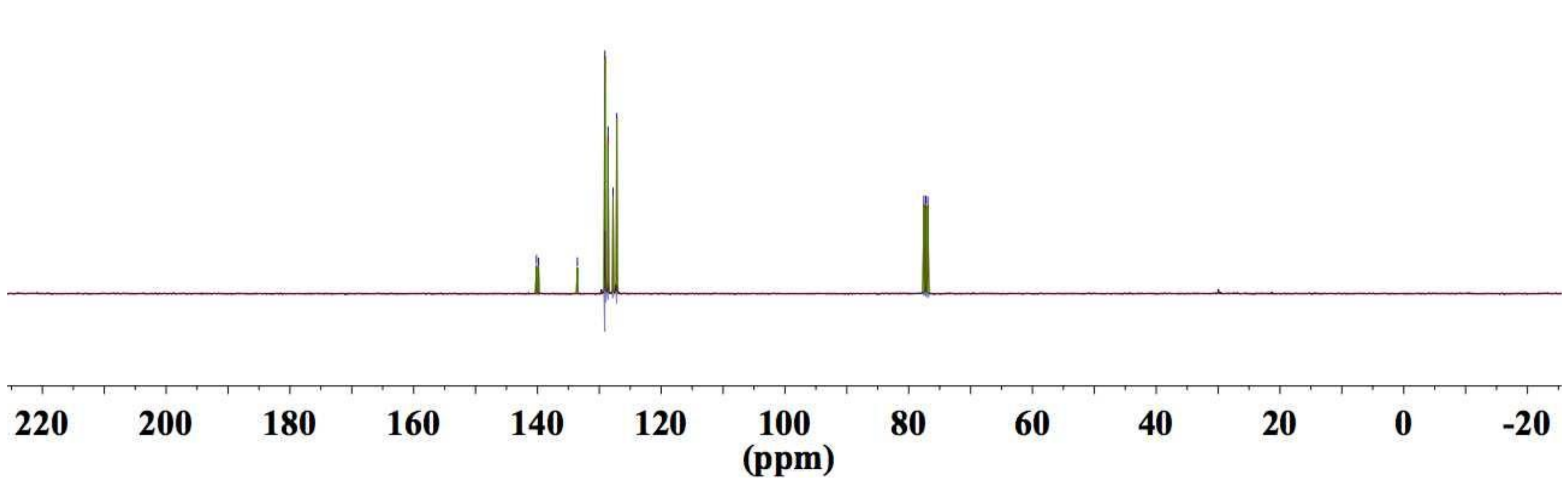
140.15
139.82
133.54
129.09
128.58
127.78
127.18
77.55
77.23
76.91
220
200
180
160
140
120
100
(ppm)
80
60
40
20
0
-20

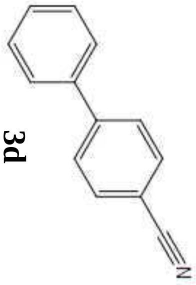

3d

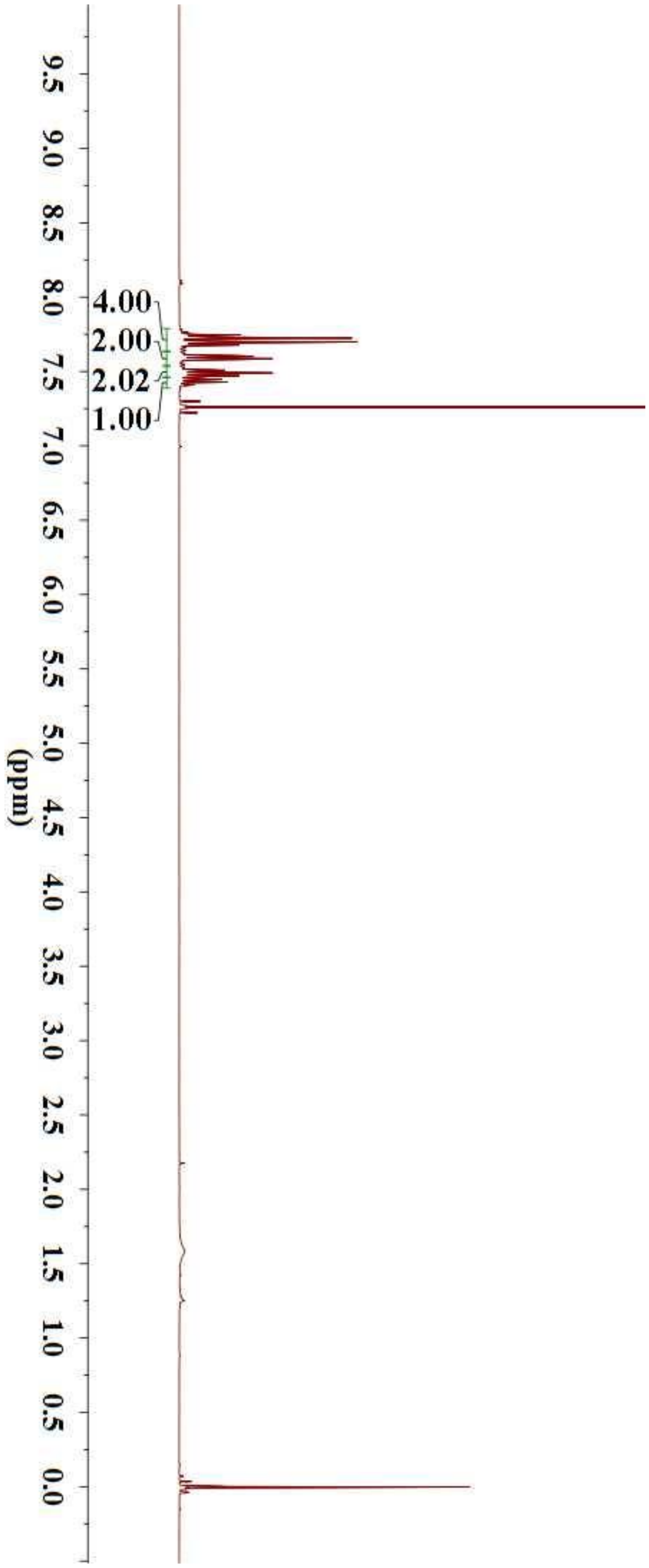
4.00
2.00
2.02
1.00
9.5 9.0 8.5 8.0 7.5 7.0 6.5 6.0 5.5 5.0 4.5 4.0 3.5 3.0 2.5 2.0 1.5 1.0 0.5 0.0
(ppm)

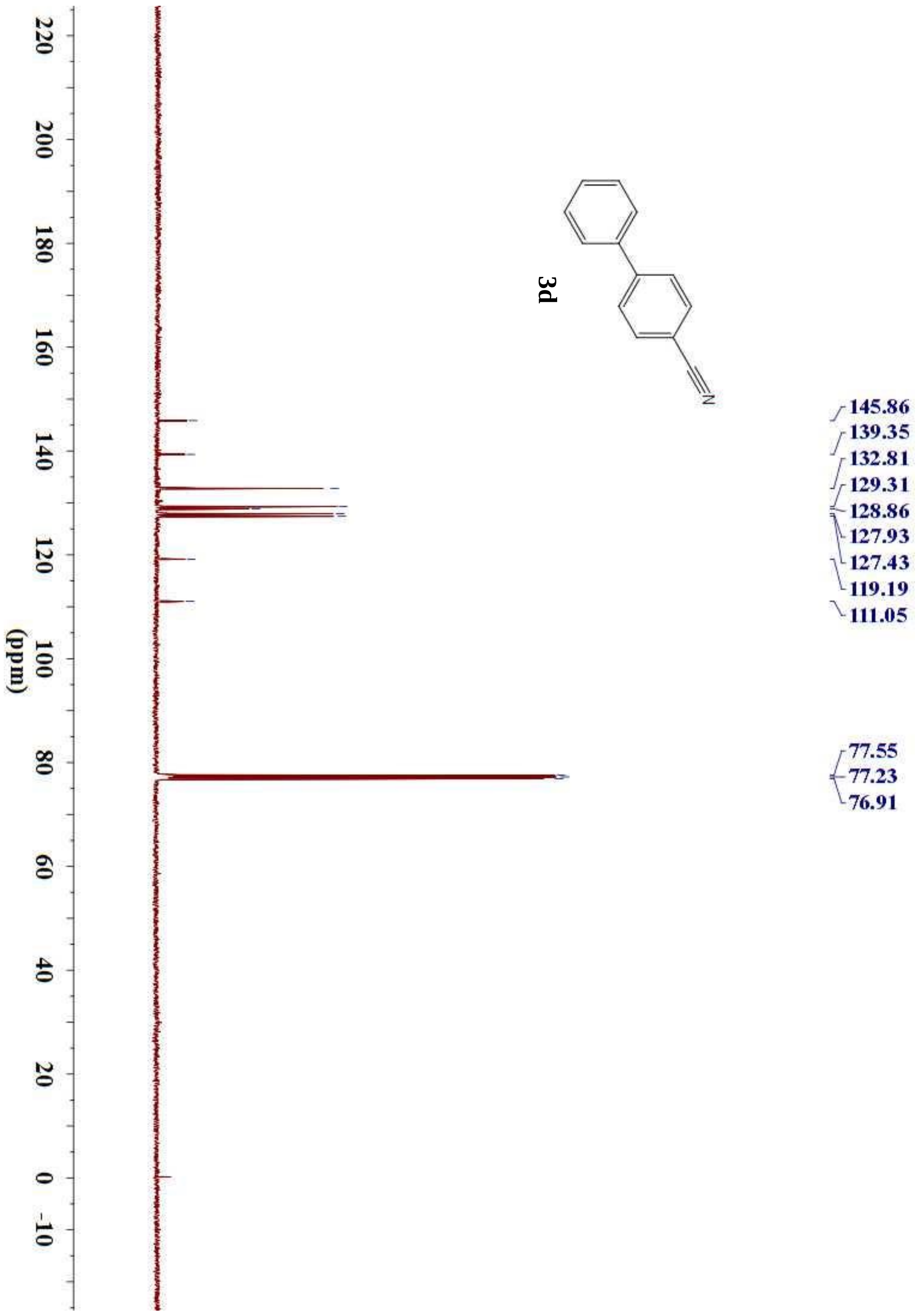
3d
145.86
139.35
132.81
129.31
128.86
127.93
127.43
119.19
111.05
77.55
77.23
76.91
(ppm)

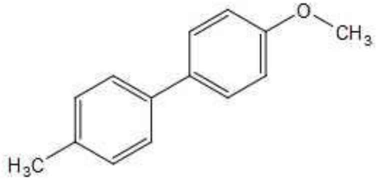
O
CH3
H3C

3e or **3h**

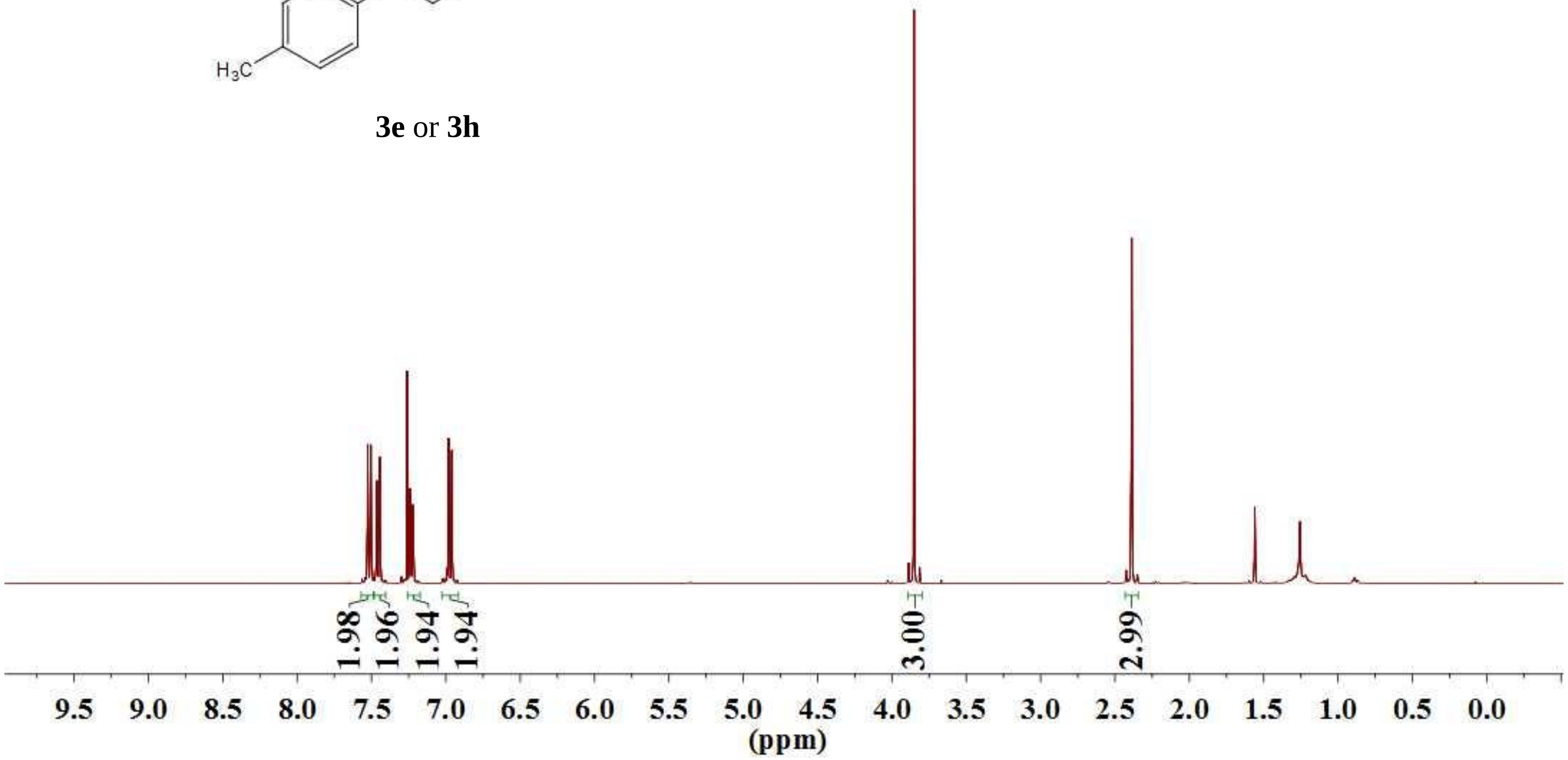
1.98
1.96
1.94
1.94
3.00
2.99
9.5 9.0 8.5 8.0 7.5 7.0 6.5 6.0 5.5 5.0 4.5 4.0 3.5 3.0 2.5 2.0 1.5 1.0 0.5 0.0
(ppm)

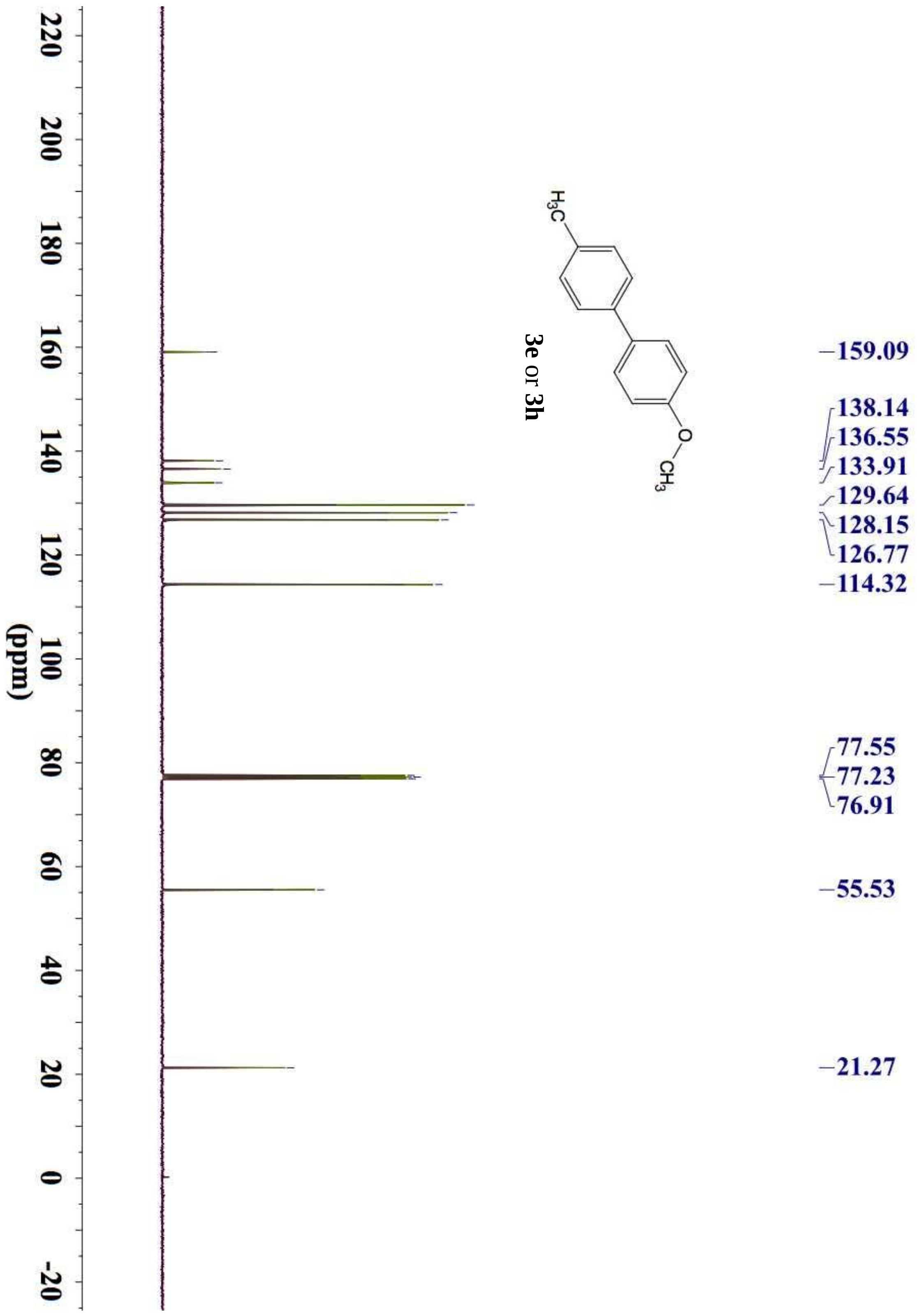

3e or 3h
159.09
138.14
136.55
133.91
129.64
128.15
126.77
114.32
77.55
77.23
76.91
55.53
21.27
220
200
180
160
140
120
100
80
60
40
20
0
-20
(ppm)

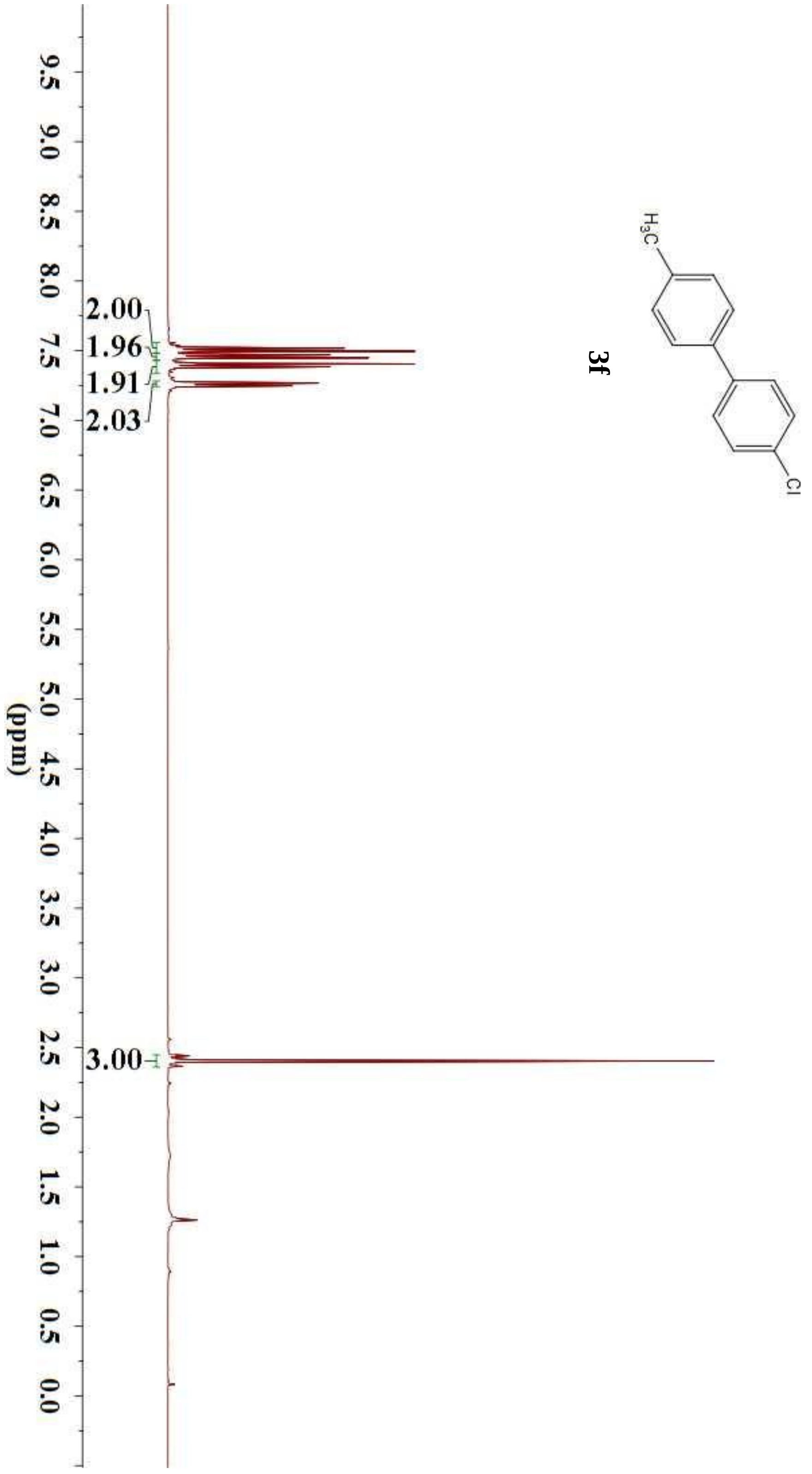
3f
H3C
Cl
2.00
1.96
1.91
2.03
3.00
9.5 9.0 8.5 8.0 7.5 7.0 6.5 6.0 5.5 5.0 4.5 4.0 3.5 3.0 2.5 2.0 1.5 1.0 0.5 0.0
(ppm)

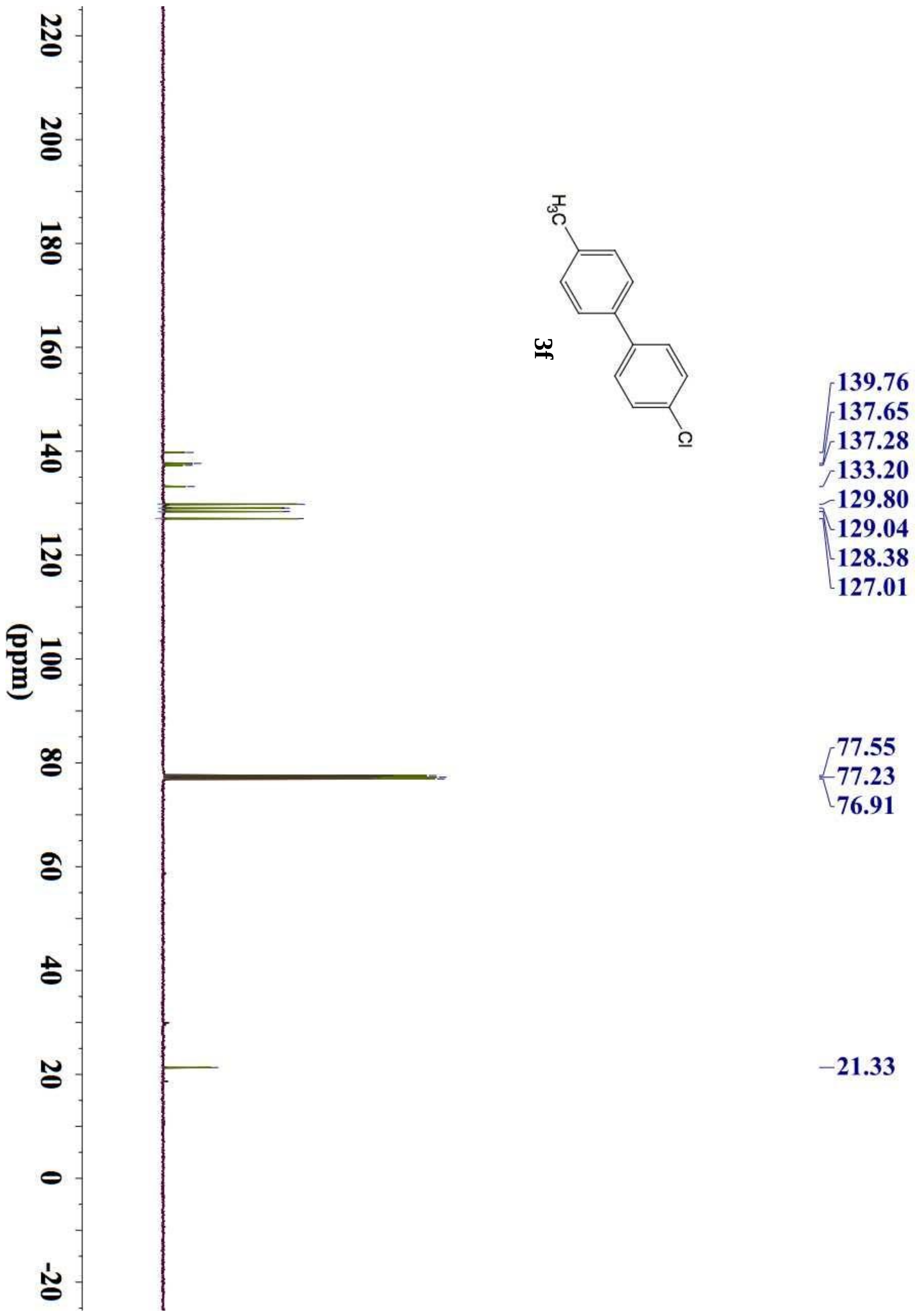

H_3C
Cl
3f
139.76
137.65
137.28
133.20
129.80
129.04
128.38
127.01
77.55
77.23
76.91
21.33
220
200
180
160
140
120
100
80
60
40
20
0
-20
(ppm)

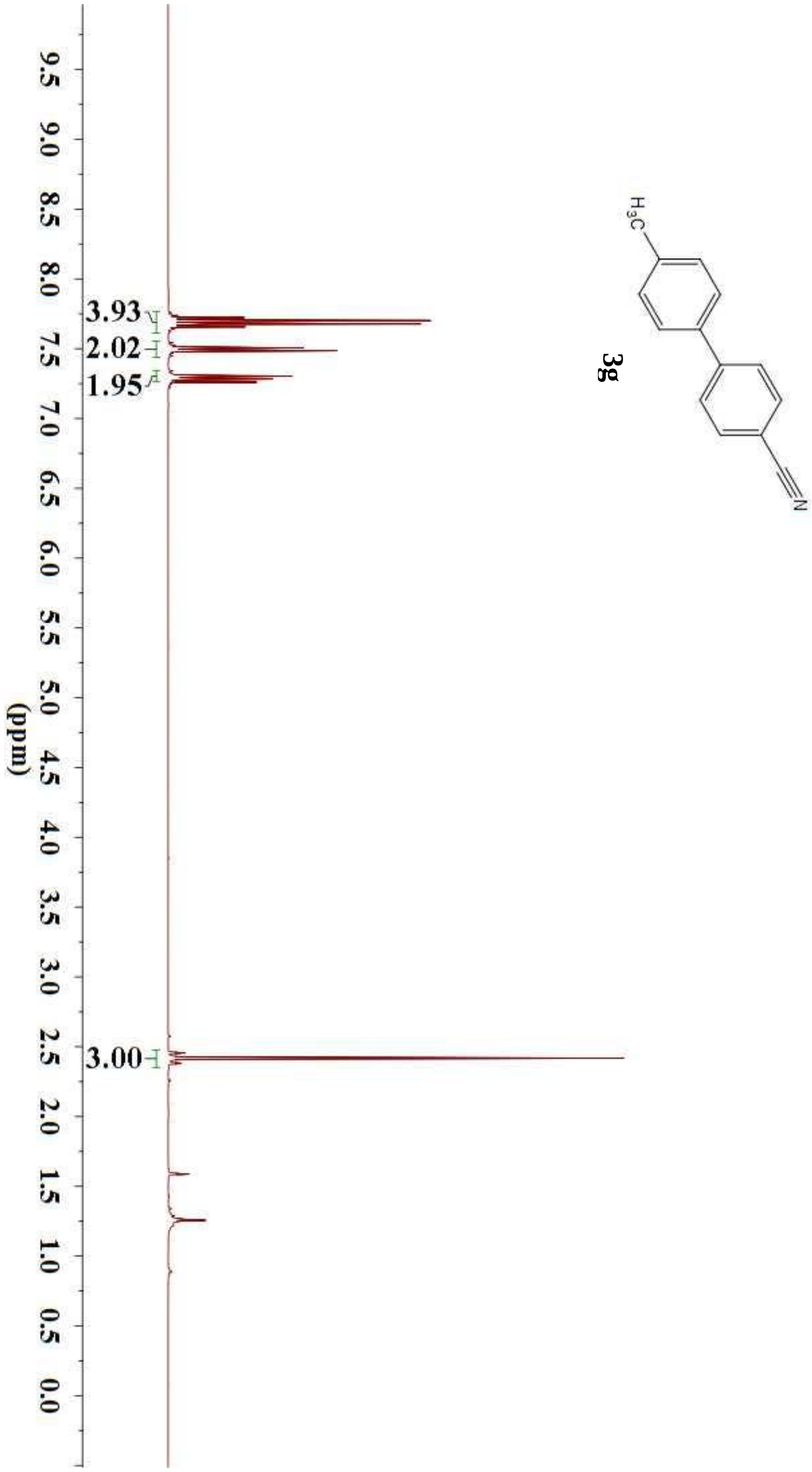
9.5
9.0
8.5
8.0
7.5
7.0
6.5
6.0
5.5
5.0
4.5
4.0
3.5
3.0
2.5
2.0
1.5
1.0
0.5
0.0
(ppm)
3.93
2.02
1.95
3.00
H_3C
N
3g

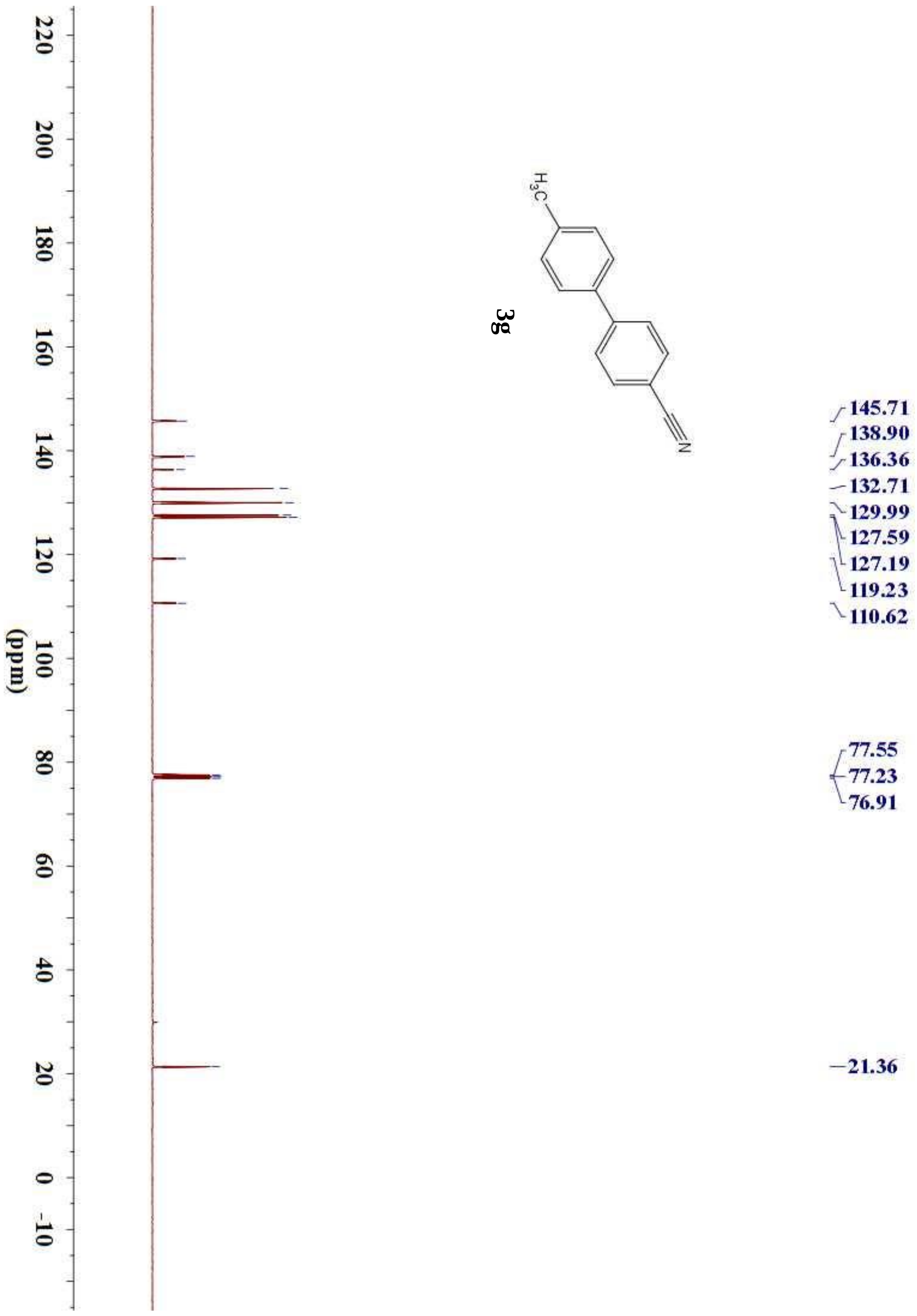
3g
H3C
N
145.71
138.90
136.36
132.71
129.99
127.59
127.19
119.23
110.62
77.55
77.23
76.91
21.36
220
200
180
160
140
120
100
(ppm)
80
60
40
20
0
-10

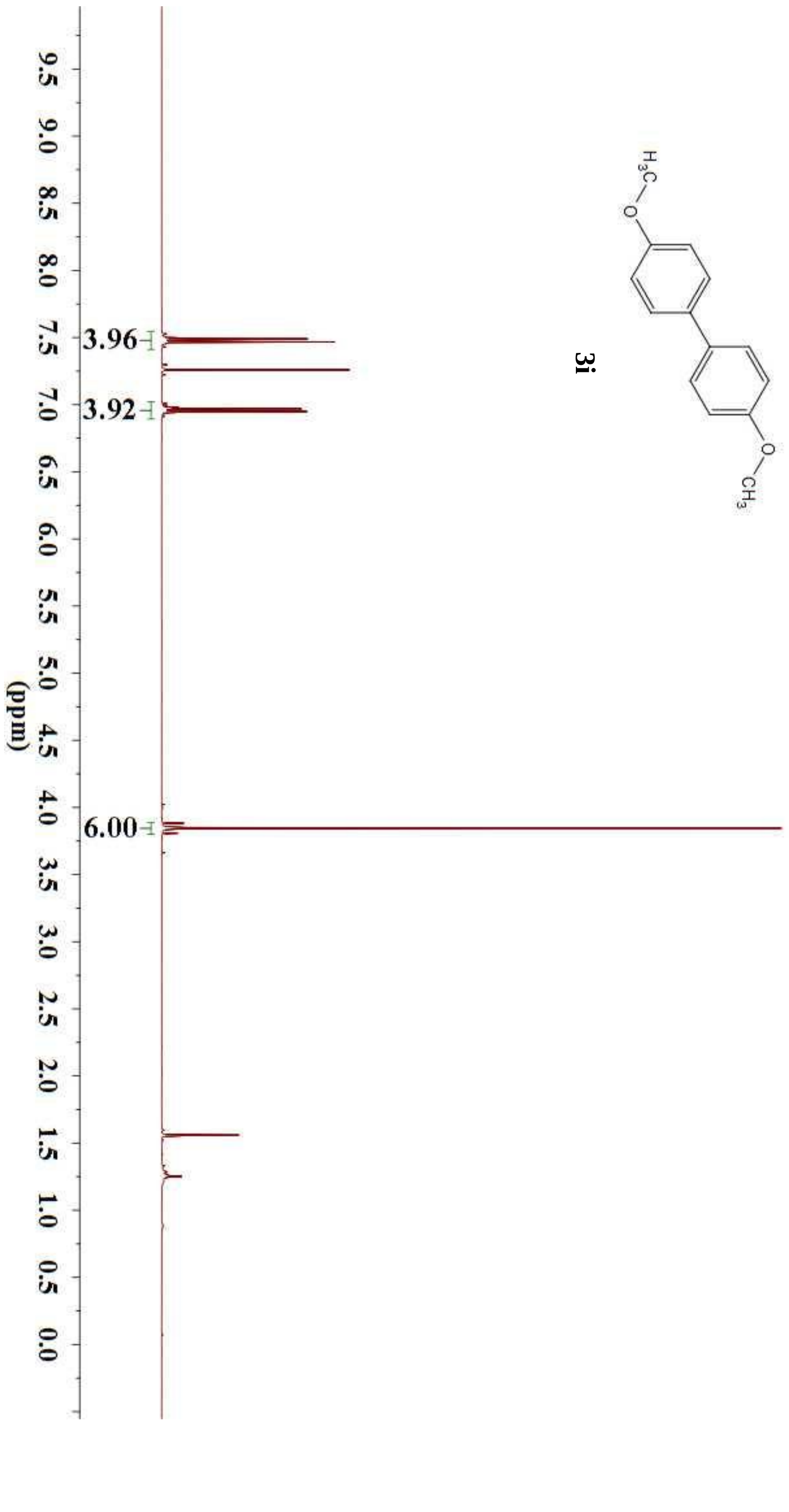

3i

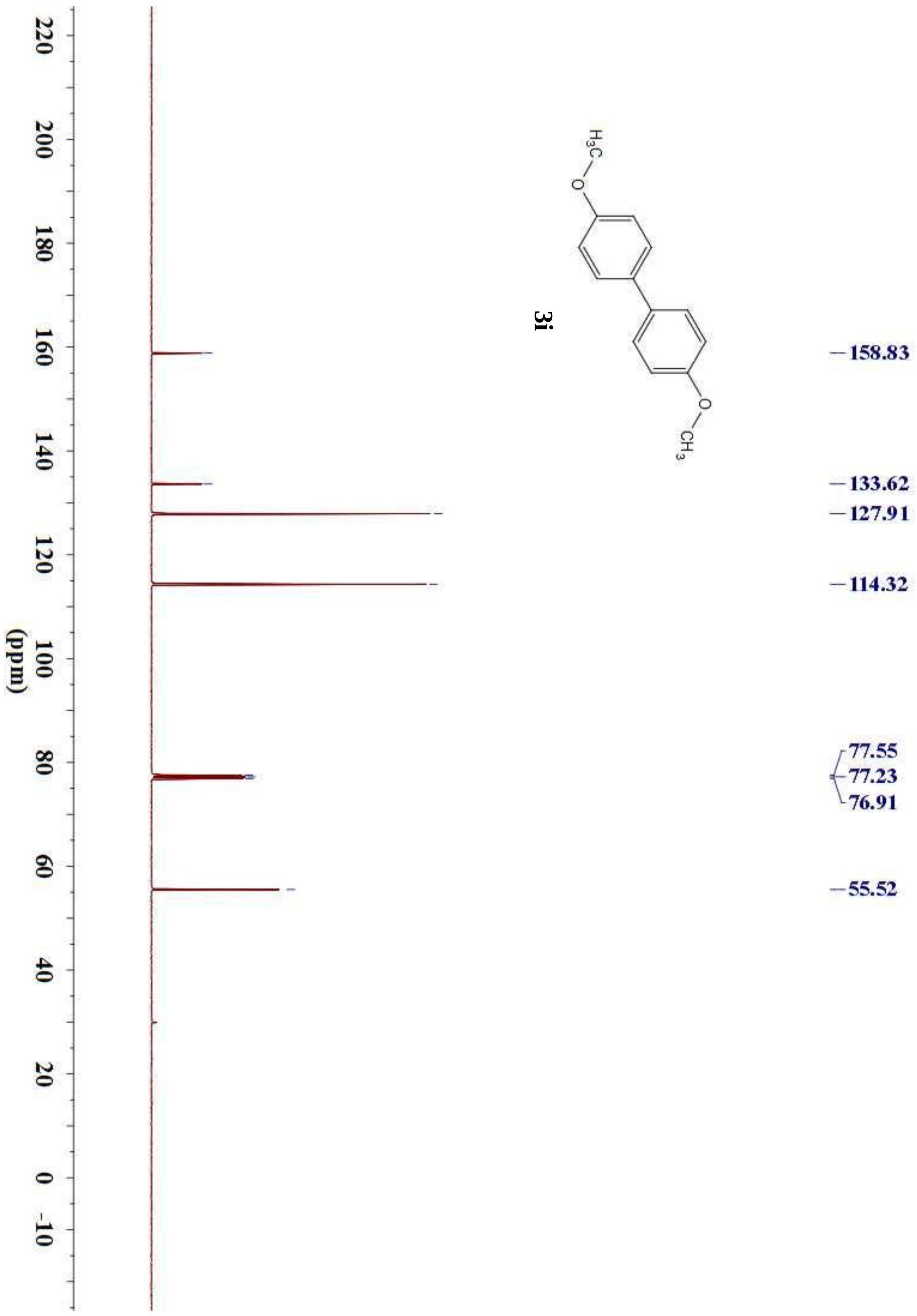
3i
H3C
O
O
CH3
158.83
133.62
127.91
114.32
77.55
77.23
76.91
55.52
220
200
180
160
140
120
100
(ppm)
80
60
40
20
0
-10

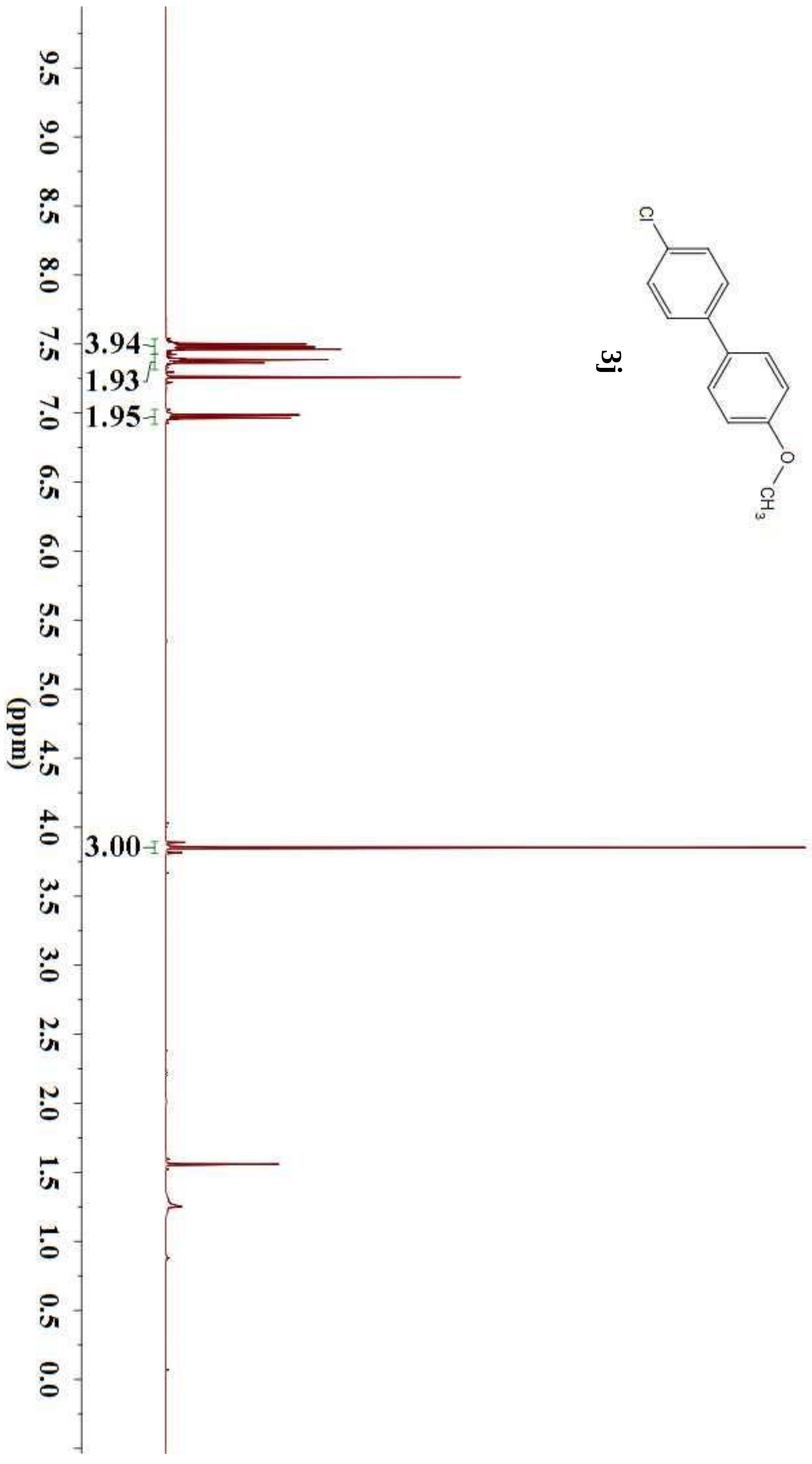

3j
Cl
O
CH3
3.94
1.93
1.95
3.00
9.5 9.0 8.5 8.0 7.5 7.0 6.5 6.0 5.5 5.0 4.5 4.0 3.5 3.0 2.5 2.0 1.5 1.0 0.5 0.0
(ppm)

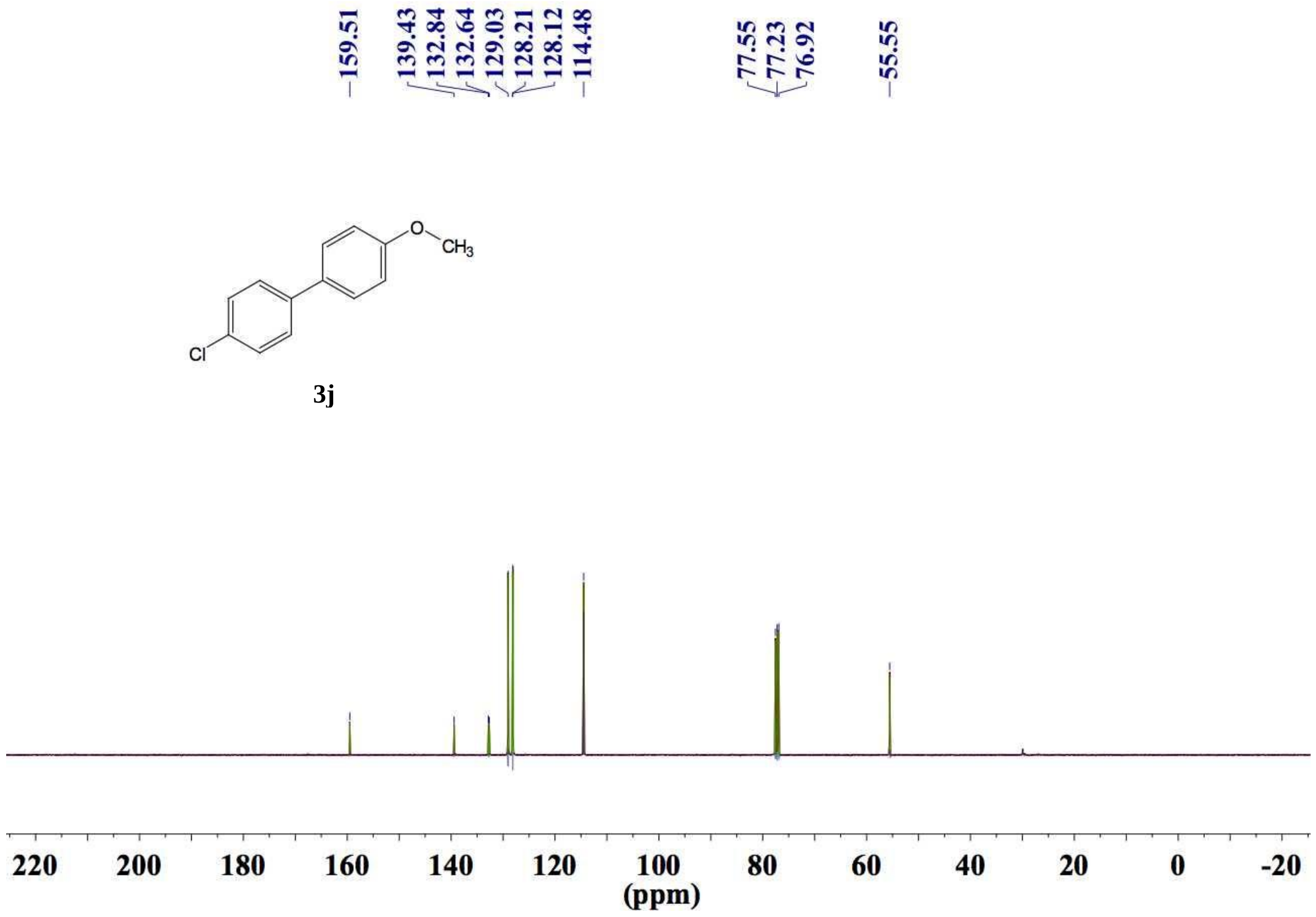
159.51
139.43
132.84
132.64
129.03
128.21
128.12
114.48
77.55
77.23
76.92
55.55
O
CH3
Cl
3j
220
200
180
160
140
120
100
(ppm)
80
60
40
20
0
-20
